# Bacteria of the Human Mouth

**Published:** 1891-10

**Authors:** E. P. Beadles


ARTICLE III.

### BACTERIA OF THE HUMAN MOUTH.

BY DR. E. P. BEADLES.

I ask the attention of the Dental Association for a few minutes to a subject which is now of great interest, not only to the minds of professional and scientific men, but one to which the great public itself is strongly taking hold. The microscope is revealing to us wonders never dreamed of.

Once in the world's history, man looked out upon this little globe of ours and considered it the center of all creation; the sun, moon and stars were minor things, created for his especial benefit. Not until the telescope came into use did men know that our earth is but a speck upon the face of creation. So small, indeed, that it would be as a mustard seed to the rocks of the mountains.

We found worlds beyond us that we knew not of; so has the microscope revealed to us worlds beneath and around us that we knew not of. Millions of living animals may be brought into existence, live and die and not occupy a space larger than that of a drop of water. Indeed, man himself in his pre-embryonic state requires the use of a strong lens to be seen. The spermatozoon is not larger than the four hundredth part of an inch in diameter, and the ovum perhaps no larger; these two infinitesmal particles of matter coming in contact in the uterus, under proper conditions, live and grow, and in time a man of full proportions of muscle, bone and brain is produced.

So the great field of the microscope is almost infinite. No professional man can afford to, in some degree, acquaint himself with the investigation of others along these lines, though the calls of everyday life prevent him from making direct investigations for himself. Indeed, it is inexcusable for a man to be treating a disease day after day, and not be, to a certain extent at least, informed as to the latest theories as well as facts regarding that disease. Hence, if there be a man before me who has not had a glance to glance over Professor Miller's "Micro-organisms of the Human Mouth," I would like to have his attention. Nothing that will follow is original; as yet I have been too busy in the general struggle for bread to make any use myself of the microscope, but I can give you, in a very condensed form, some investigations and ideas from the author above mentioned, now perhaps the leading authority on the disease germs that come under our specialty. I use as nearly as possible the language of Professor Miller's book.

### THE ORIGIN OF BACTERIA.

It has long been known that very many kinds of bacteria are found in the water we drink, the air we breath and the soil we tread upon, though the fact was not demonstrated until 1828 by Ehrenberg, although Leeuwenhock, as early as 1675, had observed microscopic organisms in the human teeth. Regarding their origin; a long and violent dispute was carried on for many years between those who claimed that they were produced spontaneously in solutions of organic matter, and those who believed that they could originate only from pre-existing germs. But in the last ten years the methods of sterilization have alone made it possible to establish the fact beyond question that the proposition *omnis cellula e cellula*, is applicable to these low forms of life, as it is to the higher animal organism.

In all cases where bacteria were found in solutions supposed to have been sterilized, it has been proven that the solutions were *not* sterilized, or that the bacteria entered from without.

Whether or not the number of species of bacteria remains constant, or whether, in the course of centuries, through the propagation of varieties, in the Darwinian sense, new species arise, it has not been settled. My own view is that they do change; basing my opinion upon the fact that all animal creation, perhaps excepting man, has changed.

THE ACTION OF OXYGEN UPON BACTERIA.

The access of atmospheric air exerts special influence upon the vegetation of bacteria. In accordance with this fact we distinguish three groups. For the development of the first, oxygen is absolutely necessary. They are called ærobic. Bacteria, of the second group, thrive better without oxygen, or even demand the absolute exclusion of air for their development. They are called anærobic. The third group flourish either with or without oxygen, at least, for a certain length of time. Most bacteria belong to the first group. Very few of the second group are known. The capability of certain germs to live and manifest their specific action without access to air, may explain their progress of tooth caries under air-tight fillings in cases where the softened denture was not thoroughly removed before inserting the filling. Acids and alkalies, especially acids, even in very dilute solutions, retard the development of bacteria. Some species, however, show important deviations from this rule; thus, for example : the acetic acid bacterium grows best in an excess of one to two per cent. of acetic acid, while micrococcus ureae will bear a high degree of alkalinity: With comparatively few exceptions, a neutral medium is best adapted to the development of bacteria.

Light, electricity and pressure have very little or no influence upon the development of bacteria. The assertion that combined fillings of gold and tin, or gold and amalgam, etc., prevent the development of bacteria, by means of the electricity produced, is entirely without scientific production.

The principle of the struggle for existence and of natural selection plays an important part, though not fully understood, in the life of bacteria. If you bring a number of different kinds of bacteria into a common culture medium, it will be found that they do not develop with equal rapidity; one kind will always attain the supremacy. If a second nutrient medicine is injected with this culture. and the operation is continued through a series of generations, we may find that at last only one kind remains, the others having been crowded out. In the human mouth this struggle for existence seems to play an important part; otherwise we should invariably expect to find a much larger number of different kinds of bacteria than are actually present. However, the attempt, to make use of this principle in therapeutics, by bringing large numbers of harmless bacteria into the human body, in order to drive out others of a pathogenic nature, has hitherto not been accompanied by the results which had been hoped for.

### ACTION OF BACTERIA UPON LIVING MATTER.

It is a belief of very old date, which found expression in the writings of Varro as early as the first century B. C., that diseases of an epidemic character are produced by some invisible living element. Not, however, until the first quarter of the present century' was the belief placed upon a scientific basis. From this time on the attention of physicians and naturalists has been constantly directed more and more to these low forms of life as the existing causes of disease. Micro organisms were found to be present in various disorders, and finally, within the last few years since the introduction of Koch's well-known culture methods, the parasitic nature of a large number of diseases has been established beyond doubt. For example, we may name anthrax, relapsing fever, abdominal typhus lepra, gonorrhœa, tuberculosis, lupus, cholera, pneumonia, syphilis, malaria, glanders, erysipelas, diphtheria, puerperal fever and suppurative process.

We distinguish bacteria according to their action upon living bodies, as *pathogenic* and *non-pathogenic*. Pathogenic are all micro-organisms which, when brought into contact with any part of a living organism, under favorable conditions, multiply and give rise to either local or general disturbances. *Non*-pathogenic are those which, when brought into the organism in large quantities, soon disappear without having produced any apparent disturbance. It has been a subject of much discussion whether or not suppuration can be produced without the presence of bacteria. As the matter now stands, it seems that certain substances under certain conditions, may produce suppuration in dogs, at least, without the presence of micro-organisms. But let us come more especially to the mouth. Leeuwenhock, the celebrated Dutch scientist, was the first to observe that microscopically small organisms exist in the human mouth in great numbers. In a treatise bearing the date of 1863, he gives descriptions and diagrams of several kinds of bacteria from the mouth. These are known as spirillum sputigenum. In later years all the species in the mouth have been classed and named. Such as "leptothrix innomeriata," "bacillus buccalis maximus," "jodococcus vaginatus," etc.

Spirillum sputigenum occurs in every mouth though in varying proportions. In mouths properly cared for, it will be found in very small quantities; in neglected mouths, however, it often exists in great numbers.

### BACTERIA FOUND IN CARIES.

No one of the many theories that have been advanced to explain the cause of decay in the teeth has been generally settled upon. Hence, it may be interesting to name several of the most prominent before setting forth Professor Miller's idea of this disease. There are said to be no less than ten, as follows:

1. Depraved juices accumulated in the teeth.

2. Disturbances of nutrition.

3. Inflammation.

4. Worms.

5. Putrefaction.

6. Chemical dissolution.

7. Parasites.

8. Electrolytic decomposition.

9. Divers causes.

10. Chemico-parasitic influences.

Galen, as early as 131 A. D., refers to "Disturbances of Nutrition" as causing decay in the teeth. A great number, even at the present day, regard inflammation as the cause of decay. Professor Miller, in a long argument, seems to clearly disprove this theory. I condense. The elements which appear in inflammation of other tissues of the animal body, cannot be found in decayed dentine. It is not possible to produce decay of the tooth, or any thing resembling decay, by means of any or all of those mechanical or chemical agents which invariably produce inflammation in other tissues. We all know that we may file, grind, saw, bore and pound the living dentine, or subject it to the action of the most irritating substances, and not produce a trace of caries. Helpless teeth and dead teeth worn on plates, or as pivots, are subject to decay in the same degree as teeth with living pulps. They also show the same microscopic changes in the structure. Again, it is possible to produce artificial changes in the dentine, or in other words, artificial decay, which, under the microscope, cannot be distinguished from natural decay.

The putrefaction theory, or that the "remains of food left between the teeth occasion decay," seems to be knocked on the head by the statement that an extracted tooth may be left in a putrefying mixture for any length of time without showing any trace of decomposition.

This paper would be much too long if I were to attempt to condense what Professor Miller says in regard to the phenomena of dental decay. He holds to the chemico-parasitic theory, i. e., that chemical action takes place first in the tooth structure, closely followed, in all cases, by micro-organism.

Before passing on to notice filling materials, let us rid ourselves of the idea, which the application of the very unscientific and unprofessional name of *bugs* to bacteria has, no doubt, tended to spread, that the parasites of the human mouth make holes in the dentine by boring into it as a worm bores into wood, or by gnawing at it as a dog gnaws at a bone. Bacteria have no apparatus for boring, nor do they have mouths or any provision for breaking off small pieces of solid substances, which they swallow whole or take up directly at any point of their periphery, after the manner of an amœba. They nourish themselves alone by substances in a state of solution, and if we present them solid substances they themselves must liquify them before they can make use of such substances. Upon this power of bacteria to liquify substances of an albuminous nature, depends the destruction of the softened dentine, in other words, the second stages of decay.

### ANTISEPTIC FILLING MATERIALS.

It seems remarkable that, while so much attention has of late years been bestowed upon the antiseptic treatment of root-canals and the employment of antiseptic materials for filling them, very little attention has been given to the subject of antiseptic materials for filling cavities of decay. Various methods may be employed for determining the antiseptic action of filling material. The following is one: A tube of ordinary nutritive gelatine is injected with a bacterium from the oral cavity, which grows rapidly at room temperature without liquifying the gelatine. The gelatine is then melted and slightly shaken, so as to distribute the fungi equally throughout the medium, and poured upon a horizontal sterilized glass plate, upon which is dropped pieces of filling material. A plate prepared in ths way, if the filling material be not antiseptic, will become clouded and opaque in the course of twenty-four hours, through the development of innumerable colonies of bacteria. If, however, the pieces of filling material possess any antiseptic power, the development of fungi in their neighborhood will be greatly retarded or wholly prevented. (Illustrated.) Most of the filling materials now in use have been tested in this way with the result that the only one which possesses this action and retains it for any length of time is copper amalgam. Not only freshly mixed fillings, but pieces of old, half-worn-out fillings and even pieces of *dentine* from teeth which had been filled with copper amalgam, invariably manifested a retarding or preventing action upon the growth of bacteria. (Illustrated.) If the filling prevents the progress of decay in softened dentine under it, or in immediate contact with it, and if it retards progress of fermentation in fine spaces (leakages) between it and the marginal wall, then it is doing a great deal toward preventing the recurrence of caries, which another filling not possessing antiseptic properties would not do. Some hold that copper amalgam does not shrink. This is probably not true, but it may be that this is no special disadvantage in the material, as a slight dampness entering causes the oxide or sulphide to more speedily form, and this permeating the tooth structure renders that hardness of the dentine always found under copper amalgam. "Pulps dying under copper amalgam fillings do not so readily decompose, owing to their becoming charged with antiseptic cupric salts," says one.

Gold amalgam, freshly mixed, has a slight retarding action on bacteria; old pieces have no effect. Oxychloride of zinc, fresh, has a marked effect; twenty-four hours old, no effect. Oxyphosphate, very slight effect. Gutta-percha and tin are completely inactive. The results obtained from gold are very peculiar and perplexing. Some preparations of gold manifest a decided retarding effect upon the development of bacteria. The antiseptic effect of Pack's pellets is particularly marked. But the antiseptic effect of gold is completely destroyed by ann aling. The antiseptic power of some non-cohesive foils perhaps account for the fact that many badly inserted fillings preserve the teeth as well as they do.

The following is a curious and interesting experiment by Professor Miller. A number of freshly extracted teeth which are extensively decayed, but so as not to expose the pulp, are partially excavated, leaving, however, a thick layer of decayed dentine. They are then filled with the different filling materials which are to be tested. The teeth are then placed in a mixture of saliva and bread and kept for three days at a temperature of 30° to 40° C. They are then removed, washed and dropped for a moment in sublimate 1-100°; then washed of the sublimate and dried well with bibulous paper. Afterward they are taken by the roots and the crowns, laid upon a small anvil, a sharp blow is struck with a hammer, the filling flies out exposing the untouched surface of the carious dentine. Now, with a sterilized spoon-shaped excavator, remove a small piece of the decay and place upon a previously-prepared plate of sterilized gelatine or other medium. The plate is then put away in a moist chamber at or near the temperature of the human body. If now the bacteria in the carious dentine have been killed by the action of the filling material, no growth will develop around it; otherwise, in the course of forty-eight or sixty-hours, we will find that the piece of dentine becomes surrounded by a growth of varying extent. The following materials were examined by this method:

1. Copper amalgam; fifteen teeth were treated as described. In not a single case did a development of bacteria take place.

2. Gold amalgam; ten teeth. In every case development of bacteria took place around the dentine.

3. Oxyphosphate; eight teeth. Development in every case.

4. Oxychloride of zinc; eight teeth. One case remained sterile, and in seven the growth was retarded to some extent.

5. Iodoform powder, one tooth. Development of bacteria unchecked.

6. Powdered sulphate of copper incorporated with cement or gutta percha, or simply strewn upon the bottom of cavity before inserting the filling; nine teeth. No trace of bacteria in any case.

Miller very justly concludes from these experiments, that copper amalgam fillings have a marked anti-bacterial influence upon the walls of the cavities containing them, and while not hasty in his conclusions, expresses himself as having great confidence in the preservative qualities of copper amalgam fillings. Certainly the facts here set forth are worthy of our most careful considerations. We can give some comfort to smokers, as the smoke of a cigar is a destroyer of bacteria. This being the case, we should expect to find no bacteria in the mouth of an habitual user of the weed, but we must remember that it is quite impossible for the smoke to penetrate all the crevices found in the oral cavity.

One word more and I am done. Professor Miller's experiments with micro-organisms and the diseases they cause, make him condemn strongly implantation and even transplantation, and I, for one, fully agree with him. I believe there is no excuse for the filthy practice of implantation, when in modern bridge-work we have something which will meet any case in which implantation would answer, and infinitely more lasting. Allow me to recommend to your consideration the book from which this paper has been written. You will peruse it with interest and with profit.—*Southern Dental Journal.*

